# 

**Published:** 1895-01

**Authors:** 


### SELECTIONS.

Article I.—Dental Legislation. By Dr. R. B. Weiser. - - 385
" II.—Calcification of Dental Pulps. By A. Rose, L. D. S. 400
" III.—Some Remarks on the Root-canal Question. By J. W. Downey, M. D., D. D. S. - - - - - 404
" IV.—Erosion of the Teeth. By D. E. Wiber, D. D. S. - 408
" V.—The Effect of Exercise Upon the Teeth. By A. Hugh Hipple, L. D. S., D. D. S. - - - - 409
" VI.—Pulp Canal Filling. By Mark G. McEelimney, D.D.S. 415

### MONTHLY SUMMARY.

Abrasions of the Skin. - - - - - - - 419

How Metals Get Tired. - - - - - - - 420

The Cause of Death from Chloroform. - - - - - 421

A Cause for Baldness. - - - - - - - 422

Root Filling. - - - - - - - - - 423

Medical Antisepsis. - - - - - - - - 424

Cold Water as an Antipyretic. - - - - - - 424

New Means of Local Anæsthesia. - - - - - - 425

Hydrotherapy in Fractures. - - - - - - 426

Uncommon Case of Salivary Calculus. - - - - - 427

Why People Become Deaf. - - - - - - - 427

Chloroform Nausea. - - - - - - - 428

Chloroform During Sleep. - - - - - - - 428

Bycicle-Riding for Women. - - - - - - 429

Food and the Teeth. - - - - - - - 430

Treatment of Apparent Death by Rhythmetical Traction Upon the Tongue. - - - - - - - - - 431

To Secure Perfect Articulation Between Natural and Substitute Teeth in Bridge-Work. - - - - - - 432

To Increase the Density and Hardness of Amalgam Fillings. - 432

